# A place for everything and everything in its place: linker histone H1 controls heterochromatic condensation through phase separation

**DOI:** 10.1093/plcell/koae032

**Published:** 2024-03-01

**Authors:** Regina Mencia

**Affiliations:** Assistant Features Editor, The Plant Cell, American Society of Plant Biologists, USA; Instituto de Agrobiotecnología del Litoral (CONICET-UNL), Cátedra de Biología Celular y Molecular, Facultad de Bioquímica y Ciencias Biológicas, Universidad Nacional del Litoral, 3000 Santa Fe, Argentina

If we take a closer look at eukaryotic nuclei, we can distinguish how chromatin arranges itself into two distinct states depending on its degree of condensation: the open, non-condensed, and active euchromatin and the highly condensed state known as heterochromatin. The tightly packed structure often forms visible heterochromatic foci (HF; see Fig.). These HF regions contain abundant transposable elements and showcase distinctive repressive epigenetic marks (histone modifications, histone variants, and DNA methylation), leading to an overall inactive state (Gibson et al. 2019; Huang et al. 2020). The proper compaction of chromatin is crucial for ensuring the correct cellular program, enabling access for gene expression, and maintaining the immobility of transposable elements, among other functions. But what forces drive the formation of these discrete and compacted heterochromatic areas? In this issue, Shengbo He, Yiming Yu, Liang Wang, and colleagues (He et al. 2024) address this question and identify the C-terminal region of the linker histone H1 as a central component regulating the formation of HF (see Fig.).

**Figure.**
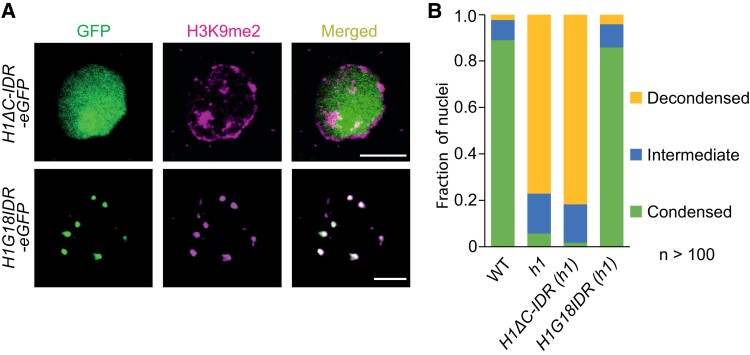
Linker histone H1 orchestrates heterocromatic foci formation through phase separation. The expression of the linker histone H1 C-terminal region with a short fragment of the DG domain (H1G18IDR) reinstates condensed HF in an *h1* background. **A)** Immunostaining. **B)** Percentages of leaf nuclei with condensed chromocenters. Scale bars, 5 *μ*m. Adapted from He et al. (2024), Figure 6.

In Arabidopsis, the natural depletion of H1 in vegetative cells, as well as in H1-knockout mutants (*h1*), leads to the dispersal of HF (He et al. 2019; Choi et al. 2020), emphasizing its pivotal role in maintaining HF integrity. Using the Hi-C method of analyzing chromatin conformation, the authors observed that decondensation of heterochromatin in *h1* mutant plants is accompanied by significant changes in the higher order of chromatin organization and increased aberrant interactions between chromosomal arms within and between chromosomes. Considering the intrinsically disordered nature of the H1 N and C-terminal domains, the authors hypothesized that the mechanism for H1-dependent HF formation involves phase separation. Both in vitro and in vivo experiments confirmed H1's capacity to undergo phase separation, contingent on the interaction with DNA, as well as the gel-like nature of the H1 condensates.

Directing their efforts toward elucidating functional regions of H1 that specifically regulate phase separation, the authors tested a wide range of constructs designed to assess the activity of each part of the H1 protein. The results provided robust evidence, both in vivo and in vitro, that the H1 C-terminal region is the key component enabling phase separation and HF formation. Moreover, the expression of the C-terminal region with a short fragment of the DG domain (H1G18IDR) not only restored condensed HF in *h1* plants but also partially restored other aspects known to be regulated by H1: the nucleosome repeat length, particularly at heterochromatic transposable elements, and DNA methylation.

Finally, paving the way for additional exploration, the authors conducted tests affirming the phase separation capability of the H1-like protein from *Bordetella pertussis* in vitro. This suggests the intriguing possibility that phase separation could be an ancient mechanism for DNA condensation, opening up an interesting path for future research. It remains to be seen whether the phase separation mechanism regulates other functions of H1 or if additional factors participate in the phase separation phenomenon by interacting with the C-terminal region.
